# In Memoriam

**DOI:** 10.2478/jofnem-2022-0051

**Published:** 2022-10-19

**Authors:** Kenneth R. Barker

**Affiliations:** 1 Department of Entomology and Plant Pathology, NC State University, Raleigh 27695, US

**Figure:**
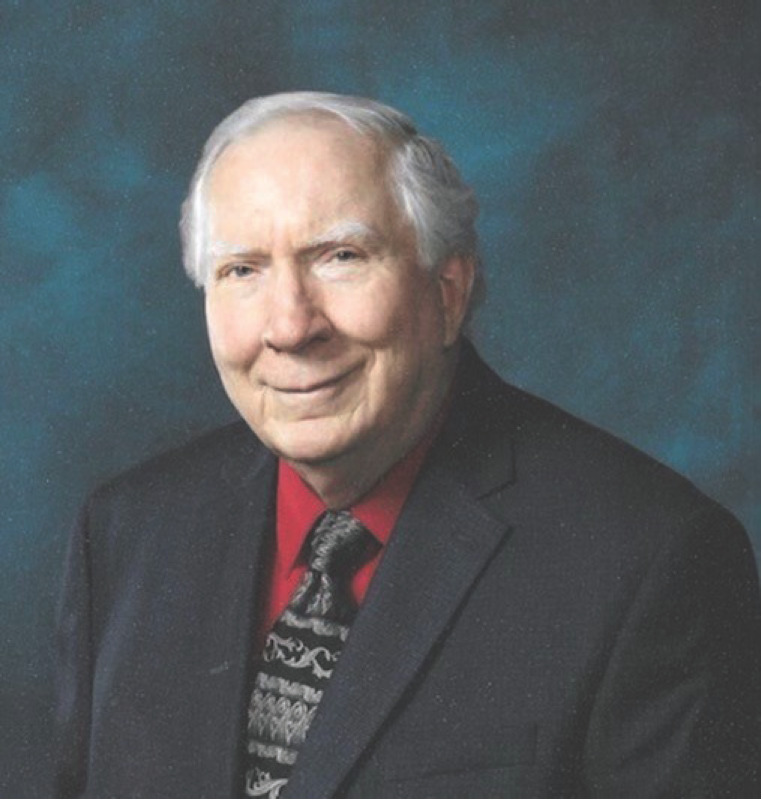


Kenneth Reece Barker passed away on August 22, 2022. Ken was born in Benham, Wilkes County, North Carolina on February 1, 1932 to Bertha Angeline Pruitt and Benjamin Harrison Barker. Ken was part of a large, loving family that ran a small tobacco farm where he quickly learned the value of hard work and the contributions of agriculture to his family and community. A visit to his high school from a North Carolina State University extension plant pathologist, Dr. Howard Garriss, convinced Ken to enroll as an undergraduate student in Agricultural Education at NC State in 1952 – Ken was the first in his family to attend college. Ken was encouraged to take a course in plant pathology taught by Dr. Arthur Kelman during his undergraduate studies at NC State, and from that point on, he was hooked. Ken was a student at NC State during the genesis of one of the premier plant nematology programs in the nation led by Dr. C.J. Nusbaum. After earning a B.S. degree in Agronomy in 1956, Ken entered a M.S. program under the mentorship of Dr. J. N. Sasser at NC State. Ken’s M.S. thesis focused on the nature of resistance to the stem and bulb nematode, *Ditylenchus dipsaci*, including investigations of variability in pathogenicity of *D. dipsaci* isolates in different host plant species.

Upon receiving his M.S. degree in Plant Pathology in 1959, Ken honored his commitment of service in the U.S. ROTC Reserves. Ken was encouraged to seek his doctoral studies at a different institution, and he chose to conduct his Ph.D. studies at the University of Wisconsin-Madison under the guidance of the famous plant pathologist, Dr. J.C. Walker. Ken studied diseases of bean and potato caused by the fungal pathogen, *Pellicularia filamentosa (Thanatephorus cucumeris)*, for his dissertation research and completed his Ph. D degree in 1961. Ken had the opportunity to conduct some special projects in nematology with Dr. Gerald Thorne during his time as a graduate student in Wisconsin, and combined with his experience in nematology at NC State, Ken was immediately offered the position of Assistant Professor at UW upon receipt of his Ph.D. Ken built a teaching and research program in nematology at UW that included nematode parasites across a range of agricultural and natural plant species.

Ken was recruited back to NC State University as a faculty member in 1966 by the Head of Plant Pathology, Dr. Don Ellis. There, Ken joined a nematology “dream team” at NC State that included C. J. Nusbaum, J. N. Sasser, Hedwig Hirschmann, Anastasios (Tasso) Triantaphyllou, and several extension faculty members with emphases in plant nematology. One of Ken’s early missions at NC State was to develop and implement a nematode advisory service in North Carolina that had been envisioned by Dr. Nusbaum. Ken established a world-class research program in the ecology, population dynamics, and management of plant-parasitic nematodes across a wide range of nematode and agricultural plant species that produced a tremendous volume of peer-reviewed journal publications, books, and book chapters. Benchmark research articles that he published with collaborators on “The interrelationships of *Meloidogyne* species with flue-cured tobacco” (*J. Nematol.* 13(1):67–79, 1981) and “Influence of planting date on population dynamics and damage potential of *Pratylenchus brachyurus* on soybean” (*J. Nematol*. 17(4):428–434, 1985) made Ken the only two-time recipient (1982 and 1986) of the Best Economic Paper Award from the Society of Nematologists. The data generated from the research of Ken and other nematologists at NC State laid the foundation of the North Carolina Nematode Assay Lab that was formalized by the NC Department of Agriculture in 1974 and currently processes greater than 50,000 samples per year that are submitted by growers, industry scientists, and agricultural professionals.

Ken served as the graduate coordinator in NC State Plant Pathology for 12 years, and he mentored numerous graduate students through his own program who graduated into careers as university faculty members, industry specialists, and government scientists. Ken considered these interactions as the most rewarding of his career, and we are fortunate that many of the scientists that he mentored have continued to make major contributions to the science of nematology and attend our professional meetings to this day. Ken became an integral member of the US AID-sponsored “International Meloidogyne Project” led by J. N. Sasser at NC State during 1975–1985 that included more than 100 participating scientists from over 70 countries worldwide. Another highlight of Ken’s career was conducting collaborative research with IMP scientists that brought him to countries around the world. Ken served as a co-editor on Volume II (Methodology) of the *Advanced Treatise on Meloidogyne* published by the IMP in 1985.

Ken served the science of nematology before and after his retirement in 1998 not only as a world-class researcher and scholar, but in multiple efforts as a member of his profession. He served as an Editor-in-Chief of the *Journal of Nematology* during 1975–1977, and as Vice-President (1978) and President (1979) of the Society of Nematologists. Ken was a founding member of the International Federation of Nematology Societies in 1996 and served as the inaugural IFNS President during 1996–2002. Ken was awarded the honor of Fellow of the Society of Nematologists (1986) and the American Phytopathological Society (1980), and he was selected as an Honorary Member of SON in 1993. Ken’s exceptional professional reputation and scholarship earned him an invitation to publish a rare prefatory chapter in the 2003 *Annual Review of Phytopathology* entitled “Perspectives in Plant and Soil Nematology” that remains relevant to this day. Ken was also invited in 2015 to prepare a video legacy of his career through NC State Libraries that provides a more candid view of his life in his own words (https://d.lib.ncsu.edu/collections/catalog/mc00449-oh-barker-20150303).

Throughout his prestigious career, Ken remained a devoted family man. He was married to his lovely wife, Betty, since 1958 and they raised two wonderful daughters, Elizabeth and Nicole. Their families grew to include four grandchildren and together they all enjoyed the beaches of North Carolina as well as attending NC State Wolfpack football and basketball games. Ken was active as a member and in leadership roles in the Raleigh Moravian Church and he particularly enjoyed participating in giving back to the community through church activities. Ken and Betty continue to give back in spirit through several generous endowments including the Kenneth and Betty Barker Student Travel Award through APS (https://www.apsnet.org/members/give-awards/donate/giving/funds/Pages/Barker.aspx), The K. R. Barker-IFNS Endowment through the SON Cobb Foundation (https://www.nematologists.org/Cobb_Endowments), and the Kenneth R. Barker Plant Pathology Graduate Endowment at NC State University (https://go.ncsu.edu/ken-barker-plant-path-grad).

